# Pathogenic GM-CSF drives functional diversification of inflammatory macrophages in autoimmune arthritis

**DOI:** 10.1126/sciadv.aec0986

**Published:** 2026-03-25

**Authors:** Hiroki Mukoyama, Yusuke Takeuchi, Daiya Ohara, Yoonha Lee, Hitomi Watanabe, Hiroki Kato, Gen Kondoh, Akio Morinobu, Keiji Hirota

**Affiliations:** ^1^ Laboratory of Integrative Biological Science, Institute for Life and Medical Sciences, Kyoto University, Kyoto, Japan.; ^2^ Department of Rheumatology and Clinical Immunology, Graduate School of Medicine, Kyoto University, Kyoto, Japan.; ^3^ The Hakubi Center for Advanced Research, Kyoto University, Kyoto, Japan.; ^4^ Institute of Cardiovascular Immunology, Medical Faculty, University Hospital Bonn, University of Bonn, Bonn, Germany.; ^5^ Graduate Institute of Biomedical Sciences, College of Medicine, China Medical University, Taichung, Taiwan.; ^6^ Cell Therapy Center, China Medical University Hospital, Taichung, Taiwan.

## Abstract

Autoimmune T cells orchestrate joint inflammation and pain in concert with synovial macrophages; however, the mechanisms governing the development and functional diversification of these macrophages remain unclear. Using a model of T helper 17 cell (T_H_17 cell)–mediated autoimmune arthritis, we show that joint-infiltrating Ly6C^hi^ monocytes in response to autoimmune T_H_17 cells, rather than resident synovial macrophages, are the primary mediators of disease pathogenesis. Granulocyte-macrophage colony-stimulating factor (GM-CSF), a critical component of the pathogenic circuit driven by arthritogenic T_H_17 cells, does not contribute to monocyte recruitment to the synovium but facilitates their subsequent differentiation into functionally distinct synovial macrophage subsets, thereby amplifying joint inflammation. Single-cell RNA sequencing identified two GM-CSF–dependent subpopulations of pathogenic synovial macrophages—Arginase-1^+^ and epithelial cell adhesion molecule (EpCAM)^+^ clusters—both expressing proinflammatory cytokines and matrix metalloproteinases. Notably, EpCAM^+^ macrophages uniquely express *Ccl17*, a pronociceptive mediator implicated in arthritic pain. Collectively, these findings delineate a GM-CSF–driven program of macrophage diversification that underpins both joint inflammation and pain, implicating this axis in the chronic activation of inflammatory and nociceptive pathways in autoimmune arthritis.

## Teaser

GM-CSF drives macrophage diversification in autoimmune arthritis.

## INTRODUCTION

Rheumatoid arthritis (RA) is the most prevalent rheumatic disease, characterized by chronic joint inflammation, persistent pain, and progressive bone destruction. Monocytes and macrophages serve as the primary sources of proinflammatory cytokines, notably tumor necrosis factor (TNF), within inflamed synovial tissues, and play pivotal roles in the amplification and maintenance of chronic joint inflammation (*1*). Recent studies have indicated that pain associated with chronic inflammation is not merely a concomitant symptom but may actively exacerbate tissue inflammation. This effect is mediated, in part, by neurotransmitters such as the calcitonin gene–related peptide (*2*, *3*). Moreover, monocytes and macrophages have been implicated not only as central mediators of inflammation but also as direct contributors to pain generation within inflamed tissues (*4*, *5*). However, the functional specialization of synovial macrophages in driving both inflammation and pain in autoimmune arthritis remains poorly characterized.

Granulocyte-macrophage colony-stimulating factor (GM-CSF) is a pleiotropic cytokine involved in the proliferation, differentiation, and activation of myeloid cells, including granulocytes, monocytes, macrophages, and dendritic cells (*6*). While GM-CSF is largely dispensable for maintaining tissue homeostasis—with a notable exception of its crucial role in the development of alveolar macrophages (*7*)—it exerts highly pathogenic functions in the context of immune responses mediated by interleukin-17 (IL-17)–producing T helper 17 cells (T_H_17 cells). This is supported by evidence from murine models of arthritis demonstrating the therapeutic efficacy of GM-CSF or GM-CSF receptor blockade (*8*–*10*), as well as from clinical trials in patients with RA (*11*–*13*). In experimental autoimmune encephalomyelitis, GM-CSF drives the pathogenicity of monocyte-derived dendritic cells (moDCs) and inflammatory macrophages (*7*, *14*, *15*). However, despite its established role as a key T_H_17 cell–driven effector cytokine, the mechanisms by which GM-CSF regulates macrophage diversification and functional specialization in autoimmune arthritis remain poorly understood.

SKG mice, a model of autoimmune arthritis resembling RA, develop arthritis following intraperitoneal injection of mannan (*16**–**18*). Arthritis can also be induced by the adoptive transfer of CD4^+^ T cells into lymphopenic recipient mice (*17**–**19*). In this model, T_H_17 cells are critical initiators of joint inflammation and subsequent arthritis progression (*18*–*20*). We have previously shown that GM-CSF produced by T_H_17 cells, fibroblast-like synoviocytes, and synovial-resident innate lymphoid cells cooperatively drives chronic joint inflammation, presumably through the activation of monocytes and macrophages (*21*).

Using the SKG mouse model of autoimmune arthritis, we demonstrate that bone marrow (BM)–derived Ly6C^hi^ monocytes are the predominant precursors of pathogenic synovial macrophages, whose recruitment and differentiation are orchestrated by arthritogenic T_H_17 cell–mediated inflammation. Single-cell RNA sequencing (scRNA-seq) analysis further revealed a GM-CSF–dependent program that drives the diversification of joint-infiltrating inflammatory monocytes into distinct synovial macrophage subsets, including Arginase-1 (Arg1)^+^ and epithelial cell adhesion molecule (EpCAM)^+^ clusters, which contribute to both joint inflammation and pain.

## RESULTS

### Circulating CCR2^+^ Ly6C^hi^ monocytes are crucial mediators of chronic joint inflammation

**Fig. 1.**
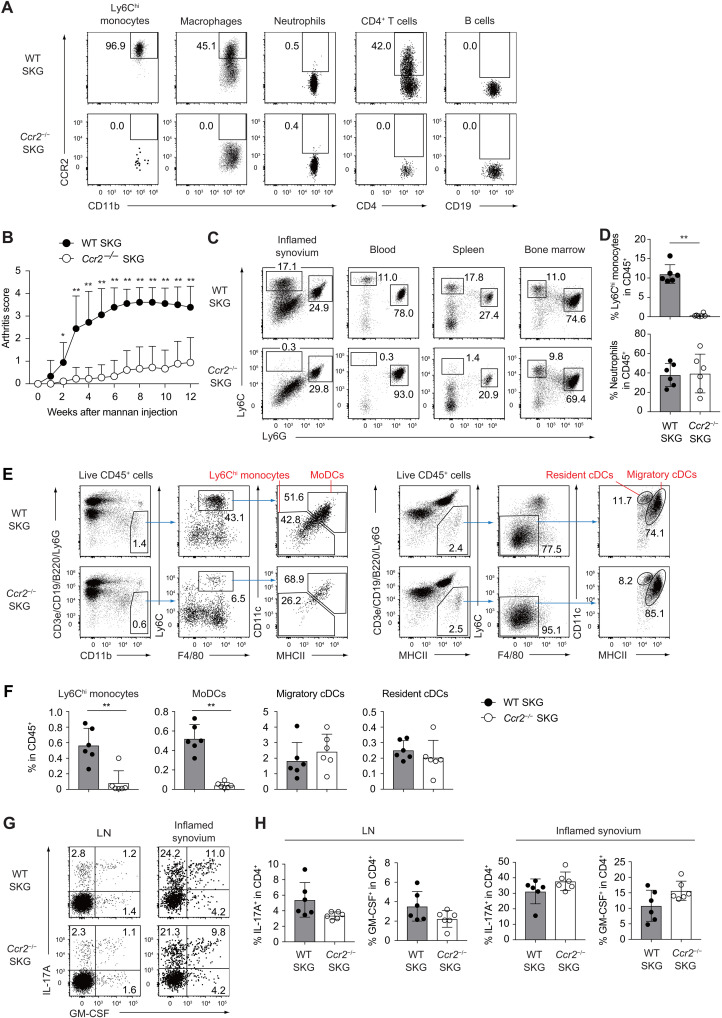
Circulating CCR2^+^ Ly6C^hi^ monocytes are crucial mediators of chronic joint inflammation. (**A**) CCR2 expression on Ly6C^hi^ monocytes, macrophages, neutrophils, CD4^+^ T cells, and B cells in the inflamed joints of WT and *Ccr2*^−/−^ SKG mice. (**B**) Arthritis scores of WT and *Ccr2*^−/−^ SKG mice (*n* = 9 each) after mannan injection (20 mg). (**C**) Flow cytometry analysis of CD45^+^ CD11b^+^ myeloid cells in inflamed synovium, blood, spleen, and BM of WT and *Ccr2*^−/−^ SKG mice. (**D**) Proportions of Ly6C^hi^ monocytes and neutrophils among synovial CD45^+^ cells of WT and *Ccr2*^−/−^ SKG mice (*n* = 6 each). (**E**) Gating strategy for Ly6C^hi^ monocytes, monocyte-derived dendritic cells (moDCs), migratory classical dendritic cells (cDCs) and resident cDCs in the popliteal lymph nodes (LNs) of WT and *Ccr2*^−/−^ SKG mice 4 weeks post–mannan injection (arthritic phase). (**F**) Percentages of Ly6C^hi^ monocytes, moDCs, and cDCs among CD45^+^ cells in the popliteal LNs of WT and *Ccr2*^−/−^ SKG mice (*n* = 6 each). (**G** and **H**) Intracellular IL-17A and GM-CSF staining of CD4^+^ T cells in the popliteal LNs and inflamed synovium of WT and *Ccr2*^−/−^ SKG mice (*n* = 6 each). *P < 0.05 and **P < 0.01. Statistical analyses were performed using two-stage step-up method of Benjamini, Krieger, and Yekutieliun (B) and Student’s *t* test (D, F, and H). Error bars denote the SD in panels. Data in (A), (C), (E), and (G) are the representatives of two independent experiments; data in (B) are pooled from three independent experiments; data in (D), (F), and (H) are pooled from two independent experiments.

We previously demonstrated that GM-CSF, a key pathogenic factor in the T_H_17 cell–mediated inflammatory circuit, is crucial for the development of autoimmune arthritis, with synovial Ly6C^hi^ monocytes acting as potential GM-CSF–responsive effectors that perpetuate chronic inflammation (*21*). To assess monocyte infiltration during disease progression, we analyzed CCR2 expression across immune cell populations in the inflamed joints of wild-type (WT) SKG and *Ccr2*^−/−^ SKG mice, which served as negative staining controls, and confirmed populations of CCR2^+^ monocytes, as well as both CCR2^+^ and CCR2^−^ macrophages, during arthritis development (Fig. 1A; see fig. S1 for gating strategies), consistent with previous reports (*22*). Notably, a subset of CD4^+^ T cells also expressed CCR2, whereas neutrophils and B cells did not.

To directly assess the functional role of CCR2 in autoimmune arthritis, we induced the disease via intraperitoneal mannan injection in WT and *Ccr2*^−/−^ SKG mice. The *Ccr2*^−/−^ SKG mice exhibited significantly attenuated arthritis scores during both the initiation and chronic phases of joint inflammation compared to the WT controls (Fig. 1B). These findings implicate CCR2^+^ monocytes, macrophages and potentially CD4^+^ T cells as key contributors to disease exacerbation. Flow cytometry analysis of Ly6C^hi^ monocytes across the BM, spleen, blood, and synovial tissue revealed that Ly6C^hi^ monocytes were virtually absent from the synovial populations of *Ccr2*^−/−^ SKG mice following arthritis induction (Fig. 1, C and D). This indicates that CCR2 expression is indispensable for the mobilization of Ly6C^hi^ monocytes from the BM into circulation and their subsequent recruitment to inflamed joints. In contrast, the trafficking of neutrophils to both the blood and joint tissues remained unaffected by CCR2 deficiency (Fig. 1, C and D).

Given that moDCs, which originate from CCR2^+^ Ly6C^hi^ monocytes in the draining lymph nodes (LNs), have been implicated in antigen presentation and in priming of T cells toward a T_H_17 cell phenotype (*7*, *14*, *15*), we hypothesized that CCR2 deficiency might impair moDC development and subsequently alter T_H_17 responses. To test this, we examined the role of moDCs in the SKG arthritis model. Notably, the frequencies of moDCs, but not classical dendritic cells (cDCs), in the draining LNs were markedly reduced in *Ccr2*^−/−^ SKG mice compared to WT controls. However, despite the reduction in moDCs, the induction of IL-17A^+^ and GM-CSF^+^ T_H_17 cells in both the draining LNs and inflamed joints remained intact 4 weeks after mannan injection (Fig. 1, E to H). These findings indicate that moDCs are not essential for T_H_17 cell differentiation or effector function in this autoimmune arthritis model.

Given that ~40% of CD4^+^ T cells expressed CCR2 and previous study shows that CCR2 mediates T_H_17 cell recruitment to inflamed tissues in experimental autoimmune encephalomyelitis (*23*), we investigated whether CCR2 expression on CD4^+^ T cells contributes to arthritis pathogenesis in the SKG model. To delineate the cell-specific roles of CCR2 in CD4^+^ T cells and myeloid cells, we adoptively transferred CD4^+^ T cells from WT or *Ccr2*^−/−^ SKG mice into either *Rag2*^−/−^ or *Ccr2*^−/−^ *Rag2*^−/−^ recipient mice (fig. S2A). Notably, *Ccr2*^−/−^ *Rag2*^−/−^ recipients exhibited significantly attenuated arthritis compared to *Rag2*^−/−^ recipients, regardless of CCR2 expression by the transferred CD4^+^ T cells, suggesting a dominant role for CCR2^+^ myeloid cells in disease pathogenesis (fig. S2B). Consistent with observations in *Ccr2*^−/−^ SKG mice, the accumulation of Ly6C^hi^ monocytes within the CD11b^+^ myeloid compartment of inflamed joints was markedly reduced in *Ccr2*^−/−^ *Rag2*^−/−^ recipients compared to *Rag2*^−/−^ controls (fig. S2, C and D). In contrast, the percentages of IL-17A^+^ and GM-CSF^+^ CD4^+^ T cells in both the draining LNs and synovial tissues were comparable across all experimental groups (fig. S2, E and F). This indicates that neither T_H_17 differentiation nor migration into inflamed joints requires CCR2 expression on T cells for arthritis development. To further dissect the cell-intrinsic requirement for CCR2 in CD4^+^ T cells, we performed a cotransfer experiment by injecting equal numbers of Thy1.1^+^ *Ccr2*^+/+^ *Il17a*^eGFP^ SKG and Thy1.2^+^ *Ccr2*^−/−^ *Il17a*^eGFP^ SKG CD4^+^ T cells into *Rag2*^−/−^ recipients (fig. S2G). Six weeks posttransfer, flow cytometry analysis revealed equivalent frequencies of *Il17a*-eGFP^+^ and GM-CSF^+^ CD4^+^ T cells in both the LNs and inflamed joints between the *Ccr2*^+/+^ and *Ccr2*^−/−^ populations (fig. S2, H and I). Collectively, these findings demonstrate that CCR2 expression is critical for the mobilization and tissue infiltration of Ly6C^hi^ monocytes that sustain chronic joint inflammation. In contrast, CCR2 expression is dispensable for T_H_17 cell differentiation, trafficking, and effector function in the context of autoimmune arthritis.

### Joint-infiltrating monocytes, but not tissue-resident macrophages, are the predominant source of pathogenic macrophages in inflamed joints

**Fig. 2.**
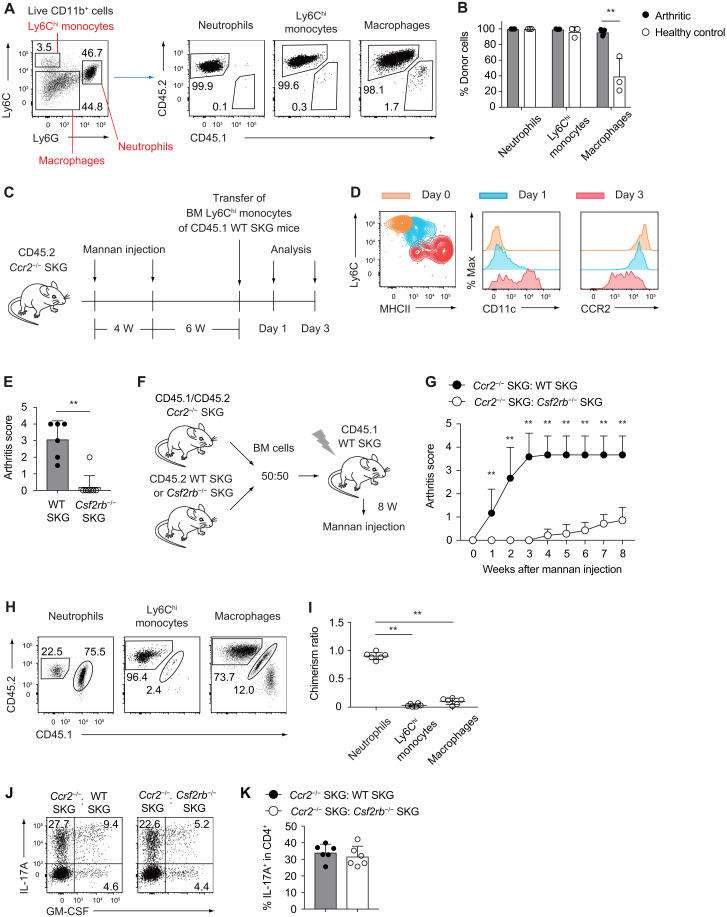
GM-CSF signaling in CCR2^+^ Ly6C^hi^ monocytes is essential for acquisition of pathogenic functions in arthritis. (**A** and **B**) Synovial tissue from BM chimeras (CD45.2 > CD45.1 SKG) was analyzed 6 weeks post–mannan injection. Mice without mannan injection were analyzed as healthy controls. Flow cytometry analysis of the CD45.1^+^ recipient– or CD45.2^+^ donor–derived cells among synovial myeloid cells. (**C**) Experimental design for the adoptive transfer of Ly6C^hi^ monocytes. W, weeks. (**D**) Expression of Ly6C, MHCII, CD11c, and CCR2 on synovial CD11b^+^ Ly6G^−^ CD45.1^+^ donor–derived cells at days 1 and 3 posttransfer. Day 0 shows pretransfer Ly6C^hi^ monocytes. (**E**) Arthritis scores of WT (*n* = 6) and *Csf2rb*^−/−^ SKG mice (*n* = 9) 12 weeks post–mannan injection. (**F**) Experimental design of mixed BM chimera reconstituted with a 1:1 mixture of BM cells from *Ccr2*^−/−^ and WT SKG mice (*n* = 6) or from *Ccr2*^−/−^ and *Csf2rb*^−/−^ SKG mice (*n* = 7). (**G**) Arthritis scores of the mixed BM chimeras shown in (F). (**H**) Flow cytometry analysis of myeloid cells in the synovium of the mixed BM chimera of *Ccr2*^−/−^ and *Csf2rb*^−/−^ SKG mice. (**I**) Contribution of CD45.1^+^ CD45.2^+^ *Ccr2*^−/−^ cells to each synovial subset, normalized to BM CD45^+^ cell chimerism. (**J** and **K**) Intracellular IL-17A and GM-CSF staining of CD4^+^ T cells in the synovium of the mixed BM chimeras (*n* = 6 each). **P < 0.01. Statistical analyses were performed using multiple *t* tests comparing arthritic and healthy control mice (B), Student’s *t* test (E and K), two-stage step-up method of Benjamini, Krieger, and Yekutieliun (G), and one-way analysis of variance (ANOVA), followed by Tukey’s multiple-comparison test (I). Error bars denote the SD in panels. Data are pooled from two (B, G, I, and K) or three (E) independent experiments; data in (A), (D), (H), and (J) are the representatives of two independent experiments.

To delineate the relative contributions of joint-infiltrating monocytes versus tissue-resident macrophages to the pool of pathogenic synovial macrophages during arthritis, we generated BM chimeras by transferring 5 × 10^6^ BM cells from CD45.2 SKG donors into lethally irradiated [6 gray (Gy)] CD45.1 SKG recipients. In healthy control chimeras, circulating neutrophils and monocytes were fully reconstituted by donor-derived (CD45.2^+^) cells, whereas a substantial fraction of synovial tissue–resident macrophages remained host derived (CD45.1^+^), reflecting their relative resistance to radiation. Following arthritis induction, however, most synovial macrophages were replaced by donor-derived CD45.2^+^ cells (Fig. 2, A and B). This suggests that infiltrating monocytes substantially outcompete or replace resident macrophages during disease progression. To directly trace the fate of infiltrating monocytes in inflamed joints, as previously reported in different tissues (*24*–*26*), we adoptively transferred sorted CD45.1^+^ Ly6C^hi^ BM monocytes into arthritic CD45.2 *Ccr2*^−/−^ SKG mice (Fig. 2C and fig. S3A). Flow cytometry analysis of the synovial cells revealed a stepwise differentiation trajectory. Ly6C^hi^ monocytes up-regulated major histocompatibility complex class II (MHCII) and CD11c as early as day 1 posttransfer, followed by progressive down-regulation of Ly6C and CCR2 by day 3, resulting in a population characterized by a Ly6C^lo^ MHCII^+^ CD11c^lo–hi^ phenotype (Fig. 2D). This phenotypic transition is consistent with the acquisition of macrophage-like characteristics in the inflamed joint microenvironment. Collectively, these findings demonstrate that joint-infiltrating Ly6C^hi^ monocytes—not radioresistant synovial tissue–resident macrophages—serve as the principal precursors of pathogenic macrophages in autoimmune arthritis.

### GM-CSF signaling in CCR2^+^ Ly6C^hi^ monocytes is essential for acquisition of pathogenic functions in arthritis

Given that joint-infiltrating monocytes and macrophages express the GM-CSF receptor (*9*), we hypothesized that GM-CSF signaling is required for Ly6C^hi^ monocytes to acquire pathogenic properties during the development of autoimmune arthritis. Consistent with this hypothesis, *Csf2rb*^−/−^ SKG mice, lacking the common β chain of the GM-CSF receptor, exhibited marked resistance to mannan-induced arthritis, phenocopying GM-CSF–deficient SKG mice (Fig. 2E) (*21*).

To directly investigate the role of GM-CSF signaling in CCR2^+^ Ly6C^hi^ monocytes, we generated mixed BM chimeras by cotransferring equal numbers of BM cells from *Ccr2*^−/−^ SKG and either WT or *Csf2rb*^−/−^ SKG mice into lethally irradiated WT SKG recipients. Arthritis was induced 8 weeks postreconstitution via mannan injection (Fig. 2F). Notably, mice that received the combination of *Ccr2*^−/−^ and *Csf2rb*^−/−^ BM cells displayed robust resistance to arthritis development, whereas control chimeras reconstituted with *Ccr2*^−/−^ and WT BM cells developed arthritis (Fig. 2G and fig. S3B). Although CD45.2^+^ monocytes and macrophages derived from *Csf2rb*^−/−^ BM donors successfully infiltrated the inflamed joints in the chimeras of *Ccr2*^−/−^ and *Csf2rb*^−/−^ BM cells, they failed to sustain or amplify joint inflammation (Fig. 2, H and I). These findings indicate that GM-CSF signaling is indispensable for Ly6C^hi^ monocytes to acquire pathogenic effector functions necessary for chronic joint inflammation. T_H_17 cell differentiation in the synovium was comparable between both chimeric groups, suggesting that GM-CSF signaling in Ly6C^hi^ monocytes and macrophages primarily contributes to the amplification of joint inflammation rather than influencing T_H_17 cell differentiation (Fig. 2, J and K).

### GM-CSF–dependent macrophage subsets exhibit distinct transcriptional profiles and developmental trajectories

**Fig. 3.**
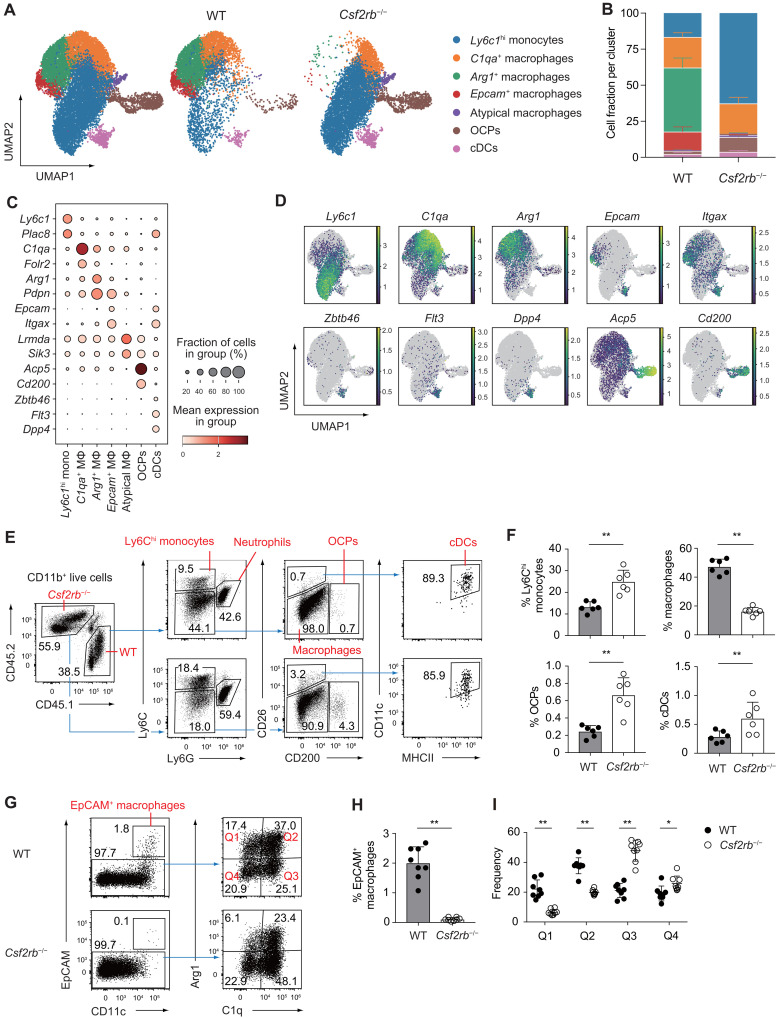
GM-CSF–dependent macrophage subsets exhibit distinct transcriptional profiles and developmental trajectories. (**A**) UMAP visualization of pooled MNPs isolated from the inflamed synovium of mixed BM chimeras generated from CD45.1 WT SKG and CD45.2 *Csf2rb*^−/−^ SKG mice (see experimental schematic in fig. S4A; gating strategy provided in fig. S4B), analyzed by scRNA-seq. Data represent merged results from four independent samples. (**B**) Relative frequency of each transcriptionally defined cluster derived from CD45.1^+^ WT and CD45.2^+^ *Csf2rb*^−/−^ MNPs. (**C** and **D**) Dot plots (C) and UMAP (D) displaying the expression of selected signature genes across MNP clusters. *Ly6c1*^hi^ mono, *Ly6c1*^hi^ monocytes; MΦ, macrophages. (**E** and **F**) Flow cytometry analysis of neutrophils, Ly6C^hi^ monocytes, macrophages, OCPs, and cDCs from CD45.1^+^ WT and CD45.2^+^ *Csf2rb*^−/−^ synovial cells in mixed BM chimeras. (F) Percentages of Ly6C^hi^ monocytes, macrophages, OCPs, and cDCs among synovial CD45.1^+^ WT and CD45.2^+^ *Csf2rb*^−/−^ CD11b^+^ cells (*n* = 6). (**G** to **I**) Flow cytometry analysis of EpCAM^+^ macrophages and intracellular staining of Arg1 and components of complement 1q (C1q), gated on CD45.1^+^ WT and CD45.2^+^ *Csf2rb*^−/−^ CD11b^+^ Ly6G^−^ Ly6C^lo^ CD26^−^ synovial macrophages from mixed BM chimeras. (H) Percentages of EpCAM^+^ macrophages within CD45.1^+^ WT and CD45.2^+^ *Csf2rb*^−/−^ CD11b^+^ Ly6G^−^ Ly6C^lo^ CD26^−^ synovial macrophages (*n* = 8). (I) Frequencies of cells among the indicated quadrants (Q1 to Q4) shown in (G) (*n* = 8). *P < 0.05 and **P < 0.01. Statistical analyses were performed using paired *t* test (F and H) and multiple *t* tests comparing CD45.1^+^ WT and CD45.2^+^ *Csf2rb*^−/−^ cells (I). Error bars denote the SD in panels. Data in (F), (H), and (I) are pooled from two independent experiments. Data in (E) and (G) are the representatives of two independent experiments.

To delineate the specific role of GM-CSF in the differentiation trajectory of Ly6C^hi^ monocytes, we generated mixed BM chimeras reconstituted with WT and *Csf2rb*^−/−^ SKG BM cells. We then performed scRNA-seq on synovial mononuclear phagocytes (MNPs) isolated 4 weeks post–arthritis induction (fig. S4A). CD11b^+^ Ly6G^−^ synovial MNPs were sorted using congenic markers CD45.1 (WT) and CD45.2 (*Csf2rb*^−/−^), yielding a dataset of 33,711 cells with expression profiles covering approximately 20,000 genes (fig. S4, A and B). Uniform manifold approximation and projection (UMAP) analysis revealed seven discrete clusters, including *Ly6c1*^hi^ monocytes, osteoclast precursor cells (OCPs), cDCs, and four macrophage subsets (Fig. 3A). In WT-derived cells, which retained responsiveness to GM-CSF signaling, *Ly6c1*^hi^ monocytes underwent differentiation into macrophage subsets characterized by *C1qa*, *Arg1*, and *Epcam* expression. In contrast, *Csf2rb*^−/−^ monocytes failed to mature beyond the *Ly6c1*^hi^ stage, with a marked absence of *Arg1*^+^ and *Epcam*^+^ macrophages (Fig. 3, A and B). These findings underscore the pivotal role of GM-CSF signaling in orchestrating the differentiation of synovial monocytes into specific macrophage subsets, particularly the *Arg1*^+^ and *Epcam*^+^ populations.

The *C1qa*^+^ macrophage cluster showed elevated expression of *Folr2*, while *Arg1*^+^ macrophages were marked by high expression of *Pdpn* (Fig. 3, C and D, and fig. S4C). *Epcam*^+^ macrophages expressed high levels of *Itgax* (CD11c) but lacked expression of cDC markers such as *Zbtb46*, *Flt3*, and *Dpp4* (*27*, *28*), indicating their macrophage identity distinct from cDC lineages. In the absence of GM-CSF signaling, *Csf2rb*^−/−^ monocytes gave rise to atypical macrophages characterized by up-regulation of *Lrmda* and *Sik3*, along with reduced expression of *Ciita*, indicating impaired MHCII expression. In addition, OCPs, defined by high expression of *Acp5*, also expressed elevated levels of *Cd200* and *Mmp9*. Flow cytometry analysis corroborated the presence of CD26 (*Dpp4*)^+^ cDCs and CD200^+^ OCPs within the synovial compartment of mixed BM chimeras (Fig. 3E and fig. S4C).

Consistent with the scRNA-seq findings, flow cytometry analysis of *Csf2rb*^−/−^ MNPs revealed higher frequencies of Ly6C^hi^ monocytes, OCPs, and cDCs than that of WT-derived cells (Fig. 3, E and F). In contrast, the proportion of mature macrophages was reduced. These data suggest that the differentiation of OCPs and cDCs proceeds independently of GM-CSF signaling (*29*–*31*). Flow cytometry analysis of CD11b^+^ Ly6G^−^ Ly6C^lo^ CD26^−^ synovial macrophages derived from *Csf2rb*^−/−^ BM confirmed the loss of EpCAM^+^ and the marked reduction of Arg1^+^ macrophages, in concordance with transcriptomic data (Fig. 3, G to I).

To further delineate the ontogeny of these macrophage subsets, we adoptively transferred Ly6C^hi^ BM monocytes from WT SKG mice into arthritic CD45.1/CD45.2 *Ccr2*^−/−^ SKG recipients. Donor-derived Ly6C^hi^ monocytes infiltrating the inflamed joints up-regulated expression of Arg1 and components of complement 1q (C1q) as early as day 1 posttransfer, followed by a progressive induction of EpCAM expression by day 6 (fig. S4, D to F). These findings demonstrate that Arg1^+^ and EpCAM^+^ macrophages originate from joint-infiltrating Ly6C^hi^ monocytes rather than from cDC precursors.

### GM-CSF signaling governs the functional diversification of inflammatory and pronociceptive synovial macrophages

**Fig. 4.**
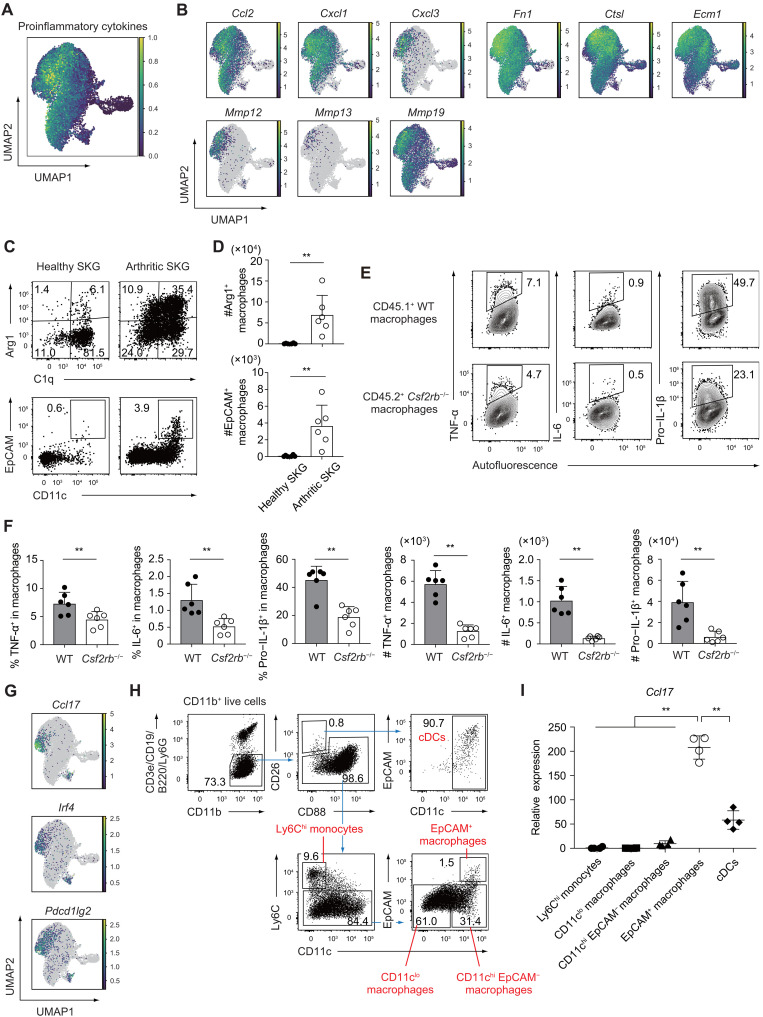
GM-CSF signaling governs the functional diversification of inflammatory and pronociceptive synovial macrophages. UMAP visualization showing the expression patterns of (**A**) key proinflammatory cytokines and (**B**) indicated genes across synovial MNPs. (**C**) Flow cytometry analysis of Arg1 and C1q expression, along with surface markers EpCAM and CD11c, within CD11b^+^ Ly6G^−^ Ly6C^lo^ CD26^−^ synovial MNPs from control (healthy) and arthritic SKG mice. Arthritic SKG mice were analyzed 4 weeks after mannan injection. (**D**) Total numbers of Arg1^+^ and EpCAM^+^ macrophages in the synovium of control and arthritic SKG mice (*n* = 6 each). (**E** and **F**) Intracellular staining of TNF-α, IL-6, and pro–IL-1β in CD11b^+^ Ly6G^−^ Ly6C^lo^ CD26^−^ CD200^−^ synovial macrophages derived from CD45.1^+^ WT and CD45.2^+^ *Csf2rb*^−/−^ cells in mixed BM chimeric mice, 4 weeks post–mannan injection. (F) Percentages and total numbers of TNF-α^+^, IL-6^+^, and pro–IL-1β^+^ macrophages, as shown in (E) (*n* = 6). (**G**) UMAP displaying the expression levels of *Ccl17*, *Irf4*, *and Pdcd1lg2*. (**H**) Gating strategy for sorting synovial cell subsets from arthritic SKG mice, including cDCs, Ly6C^hi^ monocytes, EpCAM^+^ macrophages, CD11c^lo^ macrophages, and CD11c^hi^ EpCAM^−^ macrophages from arthritic SKG mice. (**I**) Relative mRNA expression of *Ccl17* in the indicated sorted subsets (*n* = 4). Expression levels were normalized to those of *Hprt*. **P < 0.01. Statistical analyses were performed using Student’s *t* test (D), paired *t* test (F), and one-way ANOVA, followed by Tukey’s multiple-comparison test (I). Error bars denote the SD in panels. Data in (C), (E), (H), and (I) are the representatives of two independent experiments; data in (D) and (F) are pooled from two independent experiments.

Considering that GM-CSF signaling in Ly6C^hi^ monocytes and macrophages is essential for amplifying joint inflammation (Fig. 2G), we hypothesized that it also imparts pathogenic functions to synovial macrophages during their differentiation within inflamed joints. Transcriptomic profiling of GM-CSF–dependent macrophage subsets revealed elevated expression of proinflammatory cytokines and chemokines, including *Ccl2*, *Cxcl1*, and *Cxcl3*, as well as matrix metalloproteinases (MMPs; *Mmp12*, *Mmp13*, and *Mmp19*) and genes associated with an M2-like macrophage signature, as visualized in UMAP overlays (Fig. 4, A and B; fig. S4G; and table S1). These MMPs are known mediators of cartilage degradation and joint destruction (*32*–*34*). Although Arg1 expression is conventionally regarded as a hallmark of M2 macrophages with anti-inflammatory or tissue-resolving functions (*35*, *36*), these GM-CSF–driven macrophage subsets in autoimmune arthritis instead acted to sustain joint inflammation. In addition, genes associated with tissue remodeling and damage, *Fn1*, *Ctsl*, and *Ecm1*, were up-regulated in *Arg1*^+^ and *Epcam*^+^ macrophages (Fig. 4B). Fibronectin (*Fn1*) fragments have been shown to induce MMP expression or function as proteolytic mediators, contributing to cartilage degradation (*37*, *38*). Cathepsin L (*Ctsl*), a potent lysosomal protease, is highly expressed at the site of cartilage invasion (*39*), while extracellular matrix protein 1 (*Ecm1*) regulates bone remodeling and promotes angiogenesis (*40*). These findings suggest that GM-CSF drives a transcriptional program in synovial macrophages that not only amplifies local inflammation through proinflammatory cytokine production but also recruits neutrophils and Ly6C^hi^ monocytes via chemokine release, establishing a feed-forward loop that enhances joint destruction through proteolytic activity.

Supporting the transcriptomic observations, flow cytometry analysis revealed significantly increased numbers of Arg1^+^ and EpCAM^+^ macrophages in the inflamed joints of arthritic SKG mice compared to healthy controls (Fig. 4, C and D). To assess functional protein output, we examined intracellular cytokine expression in synovial macrophages from mixed BM chimeras reconstituted with CD45.1 WT and CD45.2 *Csf2rb*^−/−^ BM cells. CD11b^+^ Ly6G^−^ Ly6C^lo^ CD26^−^ CD200^−^ macrophages derived from CD45.1 WT cells exhibited significantly higher levels of TNF-α, IL-6, and pro–IL-1β compared to their CD45.2 *Csf2rb*^−/−^ counterparts (Fig. 4, E and F).

To evaluate the therapeutic impact of GM-CSF neutralization on the frequency and functional properties of synovial macrophages, we intravenously administered either an anti–GM-CSF neutralizing antibody or an isotype control antibody to SKG mice on days 0 and 3, at the time when the arthritis score reached 1.0, and analyzed macrophage subsets in inflamed synovial tissues on day 5 (fig. S5A). Although the frequencies of Arg1^+^ and EpCAM^+^ macrophages were not decreased following treatment (fig. S5, B and C), the production of pro–IL-1β, but not TNF-α or IL-6, by synovial macrophages was significantly reduced in mice receiving anti–GM-CSF antibody (fig. S5, D and E). These findings suggest that GM-CSF blockade may exert therapeutic benefit by attenuating the pro-inflammatory activity of Arg1^+^ and EpCAM^+^ macrophage subsets.

While both *Arg1*^+^ and *Epcam*^+^ macrophages expressed proinflammatory cytokines and MMPs, *Epcam*^+^ macrophages were distinguished by markedly elevated expression of a distinct gene set, including *Ccl17*, *Irf4*, and *Pdcd1lg2* (encoding programmed cell death 1 ligand 2) (Fig. 4G). Because *Ccl17* is a well-established GM-CSF–induced effector associated with nociception (*5*, *41*), we compared *Ccl17* expression across sorted synovial MNP subsets from arthritic SKG mice using reverse transcription polymerase chain reaction (RT-qPCR). Among the populations examined, EpCAM^+^ macrophages indeed exhibited the highest expression of *Ccl17*, indicating that this subset may serve as a principal effector population mediating joint pain (Fig. 4, H and I, and fig. S5F). Together, these findings demonstrate that GM-CSF signaling orchestrates the differentiation of synovial monocytes into functionally specialized synovial macrophages that perpetuate chronic inflammation, drive joint tissue destruction, and contribute to pain in autoimmune arthritis.

## DISCUSSION

Our study demonstrates that CCR2^+^ monocytes undergo a joint-specific diversification program in response to arthritogenic T_H_17 cells, shaping the functional heterogeneity of synovial macrophage subsets. The differentiation of inflammatory and pronociceptive macrophages in inflamed joints is predominantly driven by pathogenic GM-CSF and concurrently mediates chronic joint inflammation and pain.

Previous studies have reported conflicting results regarding the relative contributions of CCR2^+^ monocytes and tissue-resident macrophages to arthritis pathogenesis (*22*, *42*–*45*). These discrepancies likely arise from the specific arthritis models used. Using a T cell–dependent arthritis model, we provide clear evidence that GM-CSF drives the differentiation of highly pathogenic macrophages from CCR2^+^ monocytes, underscoring their dominant role in sustaining chronic synovitis. In contrast, tissue-resident macrophages appear unlikely to significantly contribute to disease progression, as they are displaced by infiltrating monocyte-derived macrophages during active inflammation.

Although Arg1^+^ macrophages are conventionally classified as M2 like and associated with anti-inflammatory or tissue-resolving functions (*35*, *36*), our findings indicate a contrasting role within inflamed joints. In this context, these cells expressed high levels of proinflammatory cytokines, chemokines, and MMPs, contributing to the persistence of joint inflammation and tissue destruction. These data suggest that, in T_H_17 cell–mediated autoimmune arthritis, Arg1^+^ macrophages adopt a GM-CSF–driven pathogenic program, functioning as proinflammatory effectors that sustain, rather than resolve, inflammation.

Previous studies have established that GM-CSF–induced C-C motif chemokine ligand 17 (CCL17) expression, mediated via an interferon regulatory factor 4 (IRF4)–dependent pathway, promotes nociception in murine arthritis models (*5*, *41*, *46*). Notably, a human anti-CCL17 monoclonal antibody has shown efficacy in a phase I clinical study for osteoarthritis (*47*). Using scRNA-seq and ontogeny tracing of synovial macrophages, we identified a previously unrecognized differentiation trajectory, in which infiltrating monocytes give rise to EpCAM^+^ macrophages in a GM-CSF–dependent manner. These cells highly express *Ccl17* and *Irf4*, delineating a pronociceptive macrophage subset within the inflamed synovium.

Collectively, our findings uncover a critical mechanism by which GM-CSF governs the developmental diversification of infiltrating monocytes into functionally distinct, pathogenic macrophage subsets during autoimmune arthritis. These Arg1^+^ and EpCAM^+^ macrophages amplify chronic joint inflammation and nociception. This study advances our understanding of the cellular and molecular drivers of synovial inflammation and nociception, highlighting the GM-CSF signaling axis as a promising therapeutic target for joint inflammation and pain in autoimmune diseases such as RA.

## MATERIALS AND METHODS

### Mice

SKG, *Rag2*^−/−^, and Thy1.1 congenic SKG mice used in this study were described previously (*21*). *Ccr2*^−/−^ (B6.129S4-*Ccr2^tm1Ifc^*/J), *Il17a*^eGFP^ (C57BL/6-*Il17a^tm1Bcgen^*/J), *Csf2rb*^−/−^ (B6.129S1-*Csf2rb^tm1Cgb^*/J), and CD45.1 congenic (B6.SJL-*Ptprc^a^ Pepc^b^*/BoyJ) mice were purchased from the Jackson Laboratory. These strains were backcrossed from the C57BL/6 to the BALB/c genetic background for more than eight generations. *Ccr2*^−/−^ *Rag2*^−/−^ and *Ccr2*^−/−^ *Il17a*^eGFP^ mice were generated by intercrossing the respective strains. All mice were maintained under specific pathogen–free conditions. Both male and female mice aged 8 to 12 weeks were used for all experiments. All animal procedures were approved by the Ethical Committee of the Institute for Life and Medical Sciences, Kyoto University (S-22-26-4), and conducted in accordance with institutional guidelines.

### Induction of arthritis

Arthritis was induced either by intraperitoneal injection of 20 mg of mannan (Sigma-Aldrich) in SKG mice or by intravenous adoptive transfer of CD4^+^ T cells into recipient mice (*18*, *19*). For adoptive transfer, CD4^+^ T cells were isolated from the spleen and peripheral LNs using MACS CD4 MicroBeads and an LS column (Miltenyi Biotec), following the manufacturer’s protocol. A total of 2 × 10^6^ CD4^+^ T cells was transferred intravenously via the tail vein into each recipient mouse. For experiments involving a 1:1 mixture of CD4^+^ T cells from two genotypes (Thy1.1^+^ *Ccr2*^+/+^ *Il17a*^eGFP^ SKG and Thy1.2^+^ *Ccr2*^−/−^ *Il17a*^eGFP^ SKG mice), cells were counted, mixed at a 1:1 ratio, and the composition confirmed via flow cytometry before transfer. Arthritis severity was scored as follows: 0 = no joint swelling; 0.5 = mild swelling of wrist or ankle; 1.0 = severe swelling of wrist or ankle. Scores of all four limbs (wrists and ankles) were summed to yield the total arthritis score for each mouse.

### Preparation of single-cell suspensions

Synovial tissue was carefully dissected from the ankle joints using scissors and forceps. The tissue was minced into small fragments and digested in 3 ml of RPMI 1640 supplemented with 2% fetal bovine serum (FBS; Gibco), deoxyribonuclease (DNase I; 0.1 mg/ml), and 0.3 U/ml each of collagenase I and collagenase IV (Worthington) at 37°C for 1 hour using a MACSmix tube rotator (Miltenyi Biotec). The digested tissue was subsequently filtered through a 70-μm cell strainer to obtain a single-cell suspension.

BM cells from femora were flushed using Hanks’ balanced salt solution mediun (Nacalai Tesque) supplemented with 2% FBS, 2 mM EDTA (Nacalai Tesque), and Penicillin-Streptomycin (Nacalai Tesque) and passed through a 70-μm cell strainer. Red blood cells were lysed using red blood cell lysis buffer (Sigma-Aldrich).

Spleens and popliteal LNs were minced and digested in RPMI 1640 containing 2% FBS, DNase I (0.1 mg/ml), and collagenase (0.5 mg/ml; Wako) at 37°C for 1 hour using the MACSmix tube rotator. The resulting cell suspension was filtered through a 70-μm strainer to obtain single-cell suspensions.

### Flow cytometry and cell sorting

For intracellular cytokine staining of CD4^+^ T cells, cells were restimulated for 2.5 hours at 37°C with phorbol 12-myristate 13-acetate (50 ng/ml; Sigma-Aldrich) and ionomycin (500 ng/ml; Sigma-Aldrich), in the presence of Brefeldin A (1 μg/ml; Merck) in RPMI 1640 supplemented with 5% FBS (Gibco), 2-mercaptoethanol (Gibco), GlutaMAX (Gibco), sodium pyruvate (Gibco), MEM NEAA (Gibco), and Penicillin-Streptomycin. For intracellular staining of macrophages, cells were incubated with Brefeldin A for 2 hours during enzymatic digestion. Then, the cells were fixed with 3.7% formaldehyde (Sigma-Aldrich), followed by permeabilization with 0.1% NP-40 (Nacalai Tesque) and staining with antibodies targeting intracellular cytokines and proteins. Before surface staining, single-cell suspensions were incubated with anti-mouse CD16/32 (93, BioLegend), followed by incubation with either Fixable Viability Dye eFlour 506 or Fixable Viability Dye eFlour 780 (Thermo Fisher Scientific) and subsequent staining with fluorescent dye–conjugated monoclonal antibodies against surface markers: anti-mouse Ly6C (AL-21, BD Biosciences), Ly6G (1A8, BD Biosciences), CD11b (M1/70, BioLegend), CD45 (30-F11, BioLegend), CD45.1 (A20, BioLegend), CD45.2 (104, BioLegend), CD3e (145-2C11, BioLegend), CD4 (RM4-5, BD Biosciences), TCRβ (H57-597, BD Biosciences), B220 (RA3-6B2, BioLegend), CD19 (6D5, BioLegend), CD90.1 (HIS51, eBioscience), CD90.2 (30-H12, BioLegend), CD11c (N418, BioLegend), IA/IE (M5/114.15.2, BioLegend), CCR2 (Y15-488.rMAb, BD Biosciences), CD49b (DX5, BioLegend), SiglecF (S17007L, BioLegend), CD103 (M290, BD Biosciences), EpCAM (G8.8, BioLegend), CD26 (H194-112, BioLegend), CD88 (20/70, eBioscience), CD200 (OX-90, BioLegend), and PE594-Streptavidin (BioLegend). Intracellular staining was performed with anti-mouse IL-17A (TC11-18H10.1, BD Biosciences), GM-CSF (MP1-22E9, BD Biosciences), anti-C1q (RmC7H8, BD Biosciences), anti-Arg1 (A1exF5, eBioscience), anti-mouse TNF-α (MP6-XT22, BD Biosciences), IL-6 (MP5-20F3, BioLegend), and pro–IL-1β (NJTEN3, eBioscience). Autofluorescence was detected using the KO525 channel (excitation at 405 nm; bandpass filters of 525/40 nm).

CytoFLEX S (Beckman Coulter) was used for flow cytometry analysis, and FACSAria III (BD Biosciences) and an MA900 cell sorter (Sony) were used for cell sorting. Flow cytometry data were analyzed using the FlowJo software (Tree Star Inc.).

### Generation of BM chimeras

CD45.1 WT SKG mice were lethally irradiated (6 Gy) and reconstituted with 5 × 10^6^ BM cells from CD45.2 WT SKG mice. For the generation of mixed BM chimeras, a total of 5 × 10^6^ BM cells was prepared as a 1:1 mixture from donor mice of the indicated genotypes. Before transfer, the donor cell population was analyzed by flow cytometry to confirm approximately 50:50 chimerism in CD45^+^ cells. Eight weeks posttransplantation, arthritis was induced in the reconstituted chimeras via intraperitoneal injection of mannan.

### Adoptive transfer of Ly6C^hi^ monocytes

BM was harvested from the tibiae, femora, pelvis, and humeri of WT SKG mice. Cells were stained with a cocktail of biotinylated antibodies against lineage markers: Ly6G, CD3e, CD19, B220, CD49b, SiglecF, MHCII, CD11c, CD117, and Ter119. Ly6C^hi^ monocytes were purified by depleting lineage^+^ cells using antibiotin microbeads and the magnetic-activated cell sorting (MACS) system. A total of 5 × 10^6^ Ly6C^hi^ monocytes was then transferred intravenously into arthritic *Ccr2*^−/−^ SKG mice. Cells were transferred from CD45.1 WT SKG mice to CD45.2 *Ccr2*^−/−^ SKG mice in Fig. 2 and from CD45.2 WT SKG mice to CD45.1/CD45.2 *Ccr2*^−/−^ SKG mice in fig. S4.

### Single-cell isolation for RNA-seq

Single-cell suspensions were prepared from the synovial tissue of mixed BM chimeras comprising CD45.1 WT and CD45.2 *Csf2rb*^−/−^ SKG mice (*n* = 4; including two mice each). Cells were incubated with anti-mouse CD16/32 (93, BioLegend) for 10 min at 4°C and then stained with fluorophore-conjugated antibodies for 15 min at 4°C. The samples were aliquoted into two tubes, incubated for 30 min at 4°C with two different TotalSeq anti-mouse Hashtag Antibodies (C0301 and C0302, BioLegend), and filtered through 40-μm cell strainers. A total of 25,000 CD45.1^+^ CD45.2^−^ CD11b^+^ Ly6G^−^ MNPs (TotalSeq-C0301–labeled) and CD45.2^+^ CD45.1^−^ CD11b^+^ Ly6G^−^ MNPs (TotalSeq-C0302–labeled) was sorted into RPMI 1640 containing 10% FBS. The sorted cells were mixed and processed according to the 10x Genomics protocol. Cell viability and concentration were assessed using trypan blue and light microscopy.

### scRNA-seq using the 10x genomics platform

Sorted cells were processed using the Chromium Controller (10x Genomics), following the manufacturer’s instructions. Single-cell libraries were prepared using the Chromium Next GEM Single Cell 5’ Reagent Kits v2 (10x Genomics). Libraries were sequenced on an Illumina NovaSeq 6000 platform, targeting a sequencing depth of approximately 40,000 reads per cell. Raw sequencing data were processed and converted to FASTQ files using Cell Ranger mkfastq (v7.2.0).

### Downstream analysis of 10x genomics data

Raw FASTQ files were mapped to the mm10 reference genome using Cell Ranger (v7.2.0), and both gene expression and TotalSeq Hashtag counts were quantified. The resulting filtered_feature_bc_matrix.h5 files were analyzed using Scanpy (v1.10.3) (*48*) and Python (v3.9.18) with Jupyter Notebook (v7.0.4). Cells with ≤200 detected genes and genes expressed in ≤3 cells were excluded. In addition, low-quality cells were removed on the basis of mitochondrial gene content and total expression counts. Total counts per cell were normalized using the sc.pp.normalize_per_cell function, followed by log transformation of expression values. Sample demultiplexing was performed on the basis of TotalSeq Hashtag expression to assign cell identity (CD45.1 WT SKG or CD45.2 *Csf2rb*^−/−^ SKG), and doublets were eliminated using sc.pp.scrublet. Highly variable genes were identified using sc.pp.highly_variable_genes. Clustering was performed using sc.pp.neighbors, sc.tl.umap, and sc.tl.leiden. Marker genes for each cluster were selected using sc.tl.rank_genes_groups, and gene set expression scores were calculated using sc.tl.score_genes. The full gene set is provided in table S1. These bioinformatics analyses were performed within a Singularity container (v3.8.6) to ensure reproducibility.

### GM-CSF blockade experiment

SKG mice were intravenously administered 300 μg of either an anti–GM-CSF neutralizing antibody (MP1-22E9, BioLegend) or a rat immunoglobulin G2a isotype control antibody (RTK2758, BioLegend).

### RT-qPCR of sorted cells

A total of 1000 cells from synovial cDCs, Ly6C^hi^ monocytes, EpCAM^+^ macrophages, CD11c^lo^ macrophages, and CD11c^hi^ EpCAM^−^ macrophages isolated from arthritic SKG mice was directly sorted into 500 μl of TRIzol (Thermo Fisher Scientific). Total RNA was extracted using the Direct-zol MicroPrep kit (Zymo Research) and eluted in 8 μl of nuclease-free water. cDNA was synthesized using the SuperScript VILO master mix (Thermo Fisher Scientific) and used for qPCR. Gene expression levels of *Hprt* and *Ccl17* were quantified using TaqMan probes (*Hprt*: Mm01545399_m1 and *Ccl17*: Mm01244826_g1) and THUNDERBIRD Probe qPCR Mix (TOYOBO) on a StepOnePlus Real-Time PCR System (Thermo Fisher Scientific). *Ccl17* expression levels were normalized to *Hprt* levels.

### Data presentation and statistical analysis

Data are presented as dot plots representing individual mice, with group mean ± SD indicated. Statistical analyses were performed using GraphPad Prism 7. For two-group comparisons, Student’s *t* test, paired *t* test, multiple *t* tests, and two-stage step-up method of Benjamini, Krieger, and Yekutieliun were used. For grouped data, one-way analysis of variance (ANOVA) followed by Dunn’s multiple-comparison tests or Tukey’s post hoc test was applied. *P* < 0.05 was considered statistically significant. Sample sizes are indicated in the corresponding figure legends.
