# Supplementary material for: Pathogenic GM-CSF drives functional diversification of inflammatory macrophages in autoimmune arthritis
Source: Sci Adv. 2026 Mar 25;12(13):eaec0986. doi: 10.1126/sciadv.aec0986 (PMC13015902; doi:10.1126/sciadv.aec0986)
Supplement: Supplementary file 1 — Figs. S1 to S5 Table S1 [file sciadv.aec0986_sm.pdf]

Science Advances
AAAS

Supplementary Materials for

Pathogenic GM-CSF drives functional diversification of inflammatory macrophages in autoimmune arthritis

Hiroki Mukoyama *et al.*

Corresponding author: Keiji Hirota, hkeiji@infront.kyoto-u.ac.jp

Sci. Adv. **12**, eaec0986 (2026)
DOI: 10.1126/sciadv.aec0986

This PDF file includes:

Figs. S1 to S5
Table S1

Figure S1

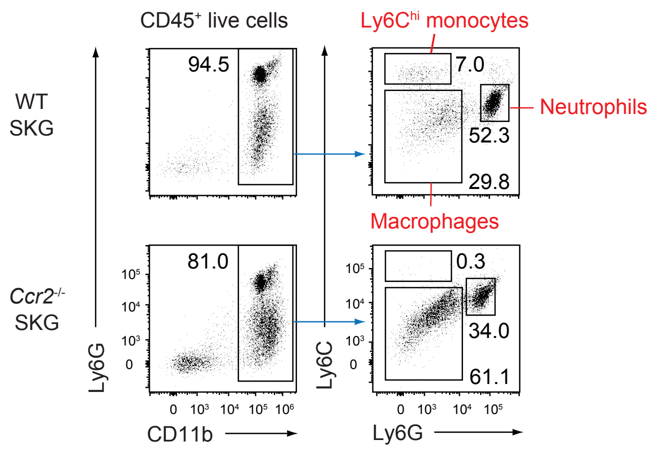

Figure S1. Gating strategy for synovial cell populations

Representative flow cytometry gating strategy used to identify Ly6C^{hi} monocytes, macrophages, and neutrophils in the synovial tissue of WT and *Ccr2*$^{-/-}$ SKG mice.

Figure S2

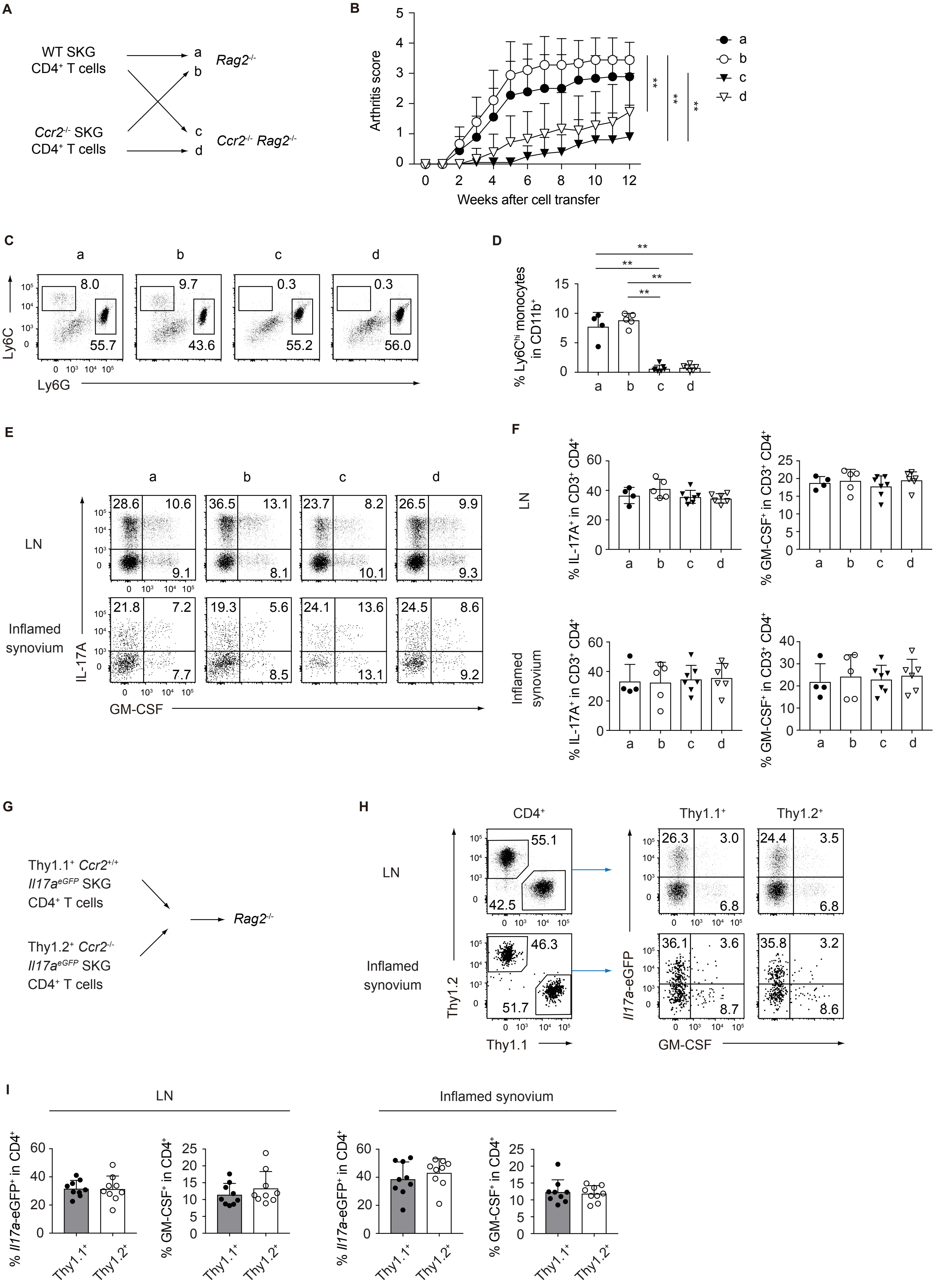

A
WT SKG CD4+ T cells
Ccr2-/- SKG CD4+ T cells
Rag2-/-
Ccr2-/- Rag2-/-
B
Arthritis score
Weeks after cell transfer
C
Ly6C
Ly6G
D
% Ly6Chi monocytes in CD11b+
E
LN
Inflamed synovium
IL-17A
GM-CSF
F
% IL-17A+ in CD3+ CD4+
% GM-CSF+ in CD3+ CD4+
G
Thy1.1+ Ccr2+/+ Il17aeGFP SKG CD4+ T cells
Thy1.2+ Ccr2-/- Il17aeGFP SKG CD4+ T cells
H
CD4+
Thy1.1+
Thy1.2+
Thy1.2
Thy1.1
Il17a-eGFP
I
LN
Inflamed synovium
% Il17a-eGFP+ in CD4+
% GM-CSF+ in CD4+

Figure S2. CCR2 expression is dispensable for Th17 differentiation, trafficking and effector functions in autoimmune arthritis.

(A) Experimental design for the adoptive transfer of $CD4^+$ T cells from WT or *Ccr2*$^{-/-}$ SKG mice into *Rag2*$^{-/-}$ or *Ccr2*$^{-/-}$*Rag2*$^{-/-}$ recipient mice. (B) Arthritis scores of the four experimental groups shown in (A) (a, n = 9; b, n = 9; c, n = 10; d, n = 9). (C, D) Flow cytometry analysis of $CD11b^+$ myeloid cells in the synovium of each group (a, n = 4; b, n = 5; c, n = 7; d, n = 6). (E, F) Intracellular IL-17A and GM-CSF staining of $CD3^+$ $CD4^+$ T cells from LNs and synovium of the four groups. (G) Experimental design for the adoptive co-transfer of $CD4^+$ T cells from Thy1.1$^+$ *Ccr2*$^{+/+}$ *Il17a*^{eGFP} SKG and Thy1.2$^+$ *Ccr2*$^{-/-}$ *Il17a*^{eGFP} SKG mice into *Rag2*$^{-/-}$ mice at a 1:1 ratio. (H) Flow cytometry analysis of $CD4^+$ T cells from LNs and synovium of *Rag2*$^{-/-}$ mice shown in (G) at 6 weeks post-transfer (arthritic phase), showing *Il17a*-eGFP expression and intracellular GM-CSF staining in Thy1.1$^+$ and Thy1.2$^+$ populations. (I) Frequencies of *Il17a*-eGFP$^+$ and GM-CSF$^+$ cells in $CD4^+$ T cells from LNs and synovium of *Rag2*$^{-/-}$ mice. Each group represents the cells derived from either Thy1.1$^+$ *Ccr2*$^{+/+}$ or Thy1.2$^+$ *Ccr2*$^{-/-}$ SKG mice (n = 9).

*$p < 0.05$, **$p < 0.01$. Statistical analyses were performed using one-way ANOVA with Dunn's multiple comparisons tests (B), one-way ANOVA with Tukey's multiple comparisons tests (D and F) and Student's t test (I). Error bars denote the SD in panel (D, F, and I). Data are pooled from three independent experiments.

Figure S3

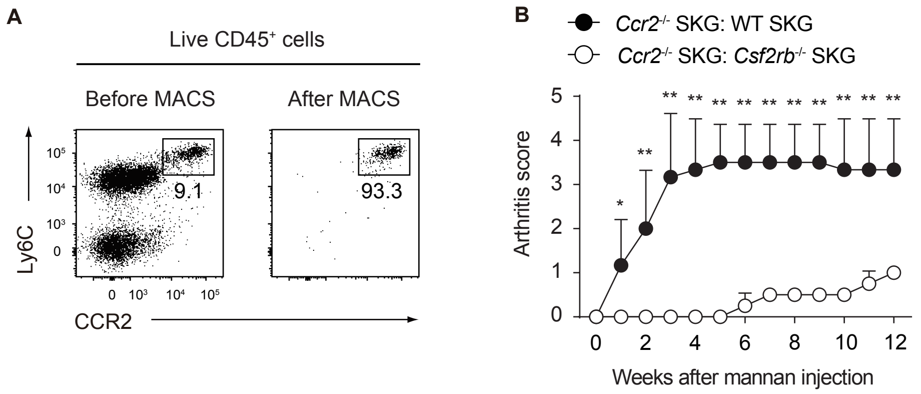

Figure S3. Purification of BM Ly6C^{hi} monocytes.

(A) Ly6C^{hi} monocytes were isolated from the BM of CD45.1 SKG mice using MACS negative selection. Lineage marker-positive cells expressing CD3e, CD19, B220, Ly6G, SiglecF, CD49b, MHCII, CD117 and Ter-119 were depleted to enrich for Ly6C^{hi} monocytes. Flow cytometry analysis shows the frequencies of Ly6C^{hi} CCR2^{+} monocytes before and after MACS enrichment. (B) Arthritis scores of mixed BM chimeras shown in Fig. 2F: *Ccr2*$^{-/-}$ + WT SKG (n = 3) and *Ccr2*$^{-/-}$ + *Csf2rb*$^{-/-}$ SKG (n = 3).

*$p < 0.05$, **$p < 0.01$. Statistical analyses were performed using two-stage step-up method of Benjamini, Krieger, and Yekutieliun (B). Error bars denote the SD in panels.

Figure S4

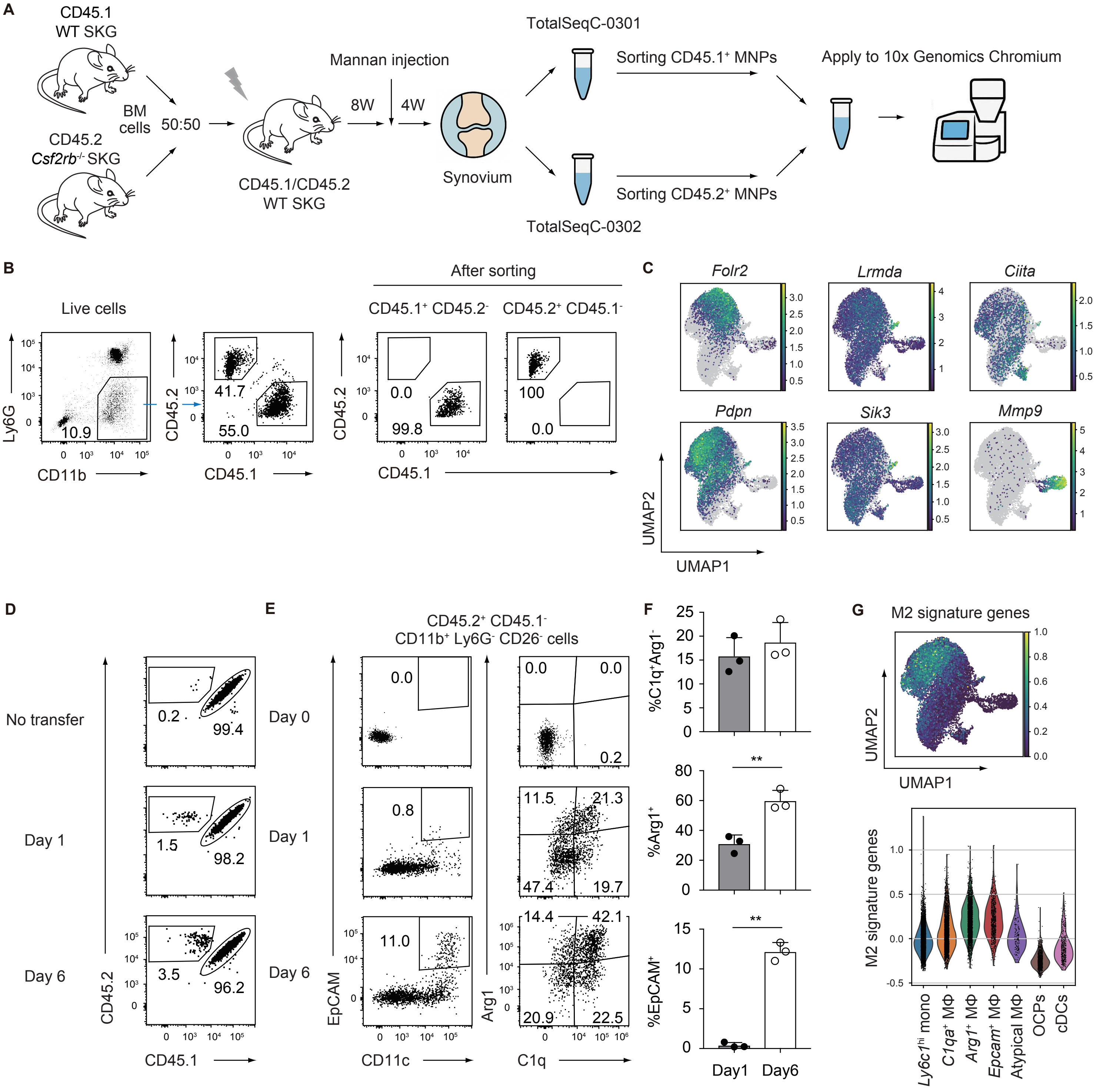

Figure S4. Arg1^{+} and EpCAM^{+} synovial macrophages originate from Ly6C^{hi} monocytes.

(A) Experimental workflow for scRNA-seq of MNPs isolated from the inflamed synovium of mixed BM chimeras generated from CD45.1 WT SKG and CD45.2 *Csf2rb*$^{-/-}$ SKG BM cells. Synovial cells were stained with fluorophore-conjugated antibodies and labeled with two distinct TotalSeq anti-mouse Hashtag Antibodies (C0301 and C0302). CD11b^{+} Ly6G^{-} MNPs were sorted as either CD45.1^{+} CD45.2^{-} (from the C0301-labeled sample) or CD45.2^{+} CD45.1^{-} (from the C0302-labeled sample), pooled, and subjected to 10x Genomics Chromium scRNA-seq. (B) Representative flow cytometry plots depicting the gating strategy used to sort CD45.1^{+} CD45.2^{-} and CD45.2^{+} CD45.1^{-} CD11b^{+} Ly6G^{-} MNPs from the inflamed synovium of the mixed BM chimeras. Sorting purity was validated and is shown in the right panels. (C) Expression of selected marker genes overlaid on the UMAP shown in Fig. 3A. (D) Ly6C^{hi} monocytes isolated from the BM of CD45.2 WT SKG mice were adoptively

transferred into arthritic CD45.1/CD45.2 *Ccr2*$^{-/-}$ SKG mice. Mice were sacrificed on day 1 and 6 post-transfer. Flow cytometry analysis showing the frequencies of CD45.2^{+} CD45.1^{-} donor-derived or CD45.2^{+} CD45.1^{+} recipient-derived cells within synovial CD11b^{+} Ly6G^{-} populations. (E, F) Flow cytometry analysis of donor-derived CD45.2^{+} CD45.1^{-} CD11b^{+} Ly6G^{-} CD26^{-} monocytes and macrophages, indicating the frequencies of C1q^{+} Arg1^{-}, Arg1^{+}, and EpCAM^{+} synovial macrophages (n = 3). Day 0 corresponds to pre-transfer Ly6C^{hi} monocytes. (G) Expression of M2-like macrophage signature genes on the UMAP shown in Fig. 3A and violin plots showing their expression across the indicated cell subsets.

**p < 0.01. Statistical analyses were performed using Student' s t test (F). Error bars denote the SD in panels. Data in (D and E) are representatives; data in (F) are pooled from two independent experiments.

Figure S5

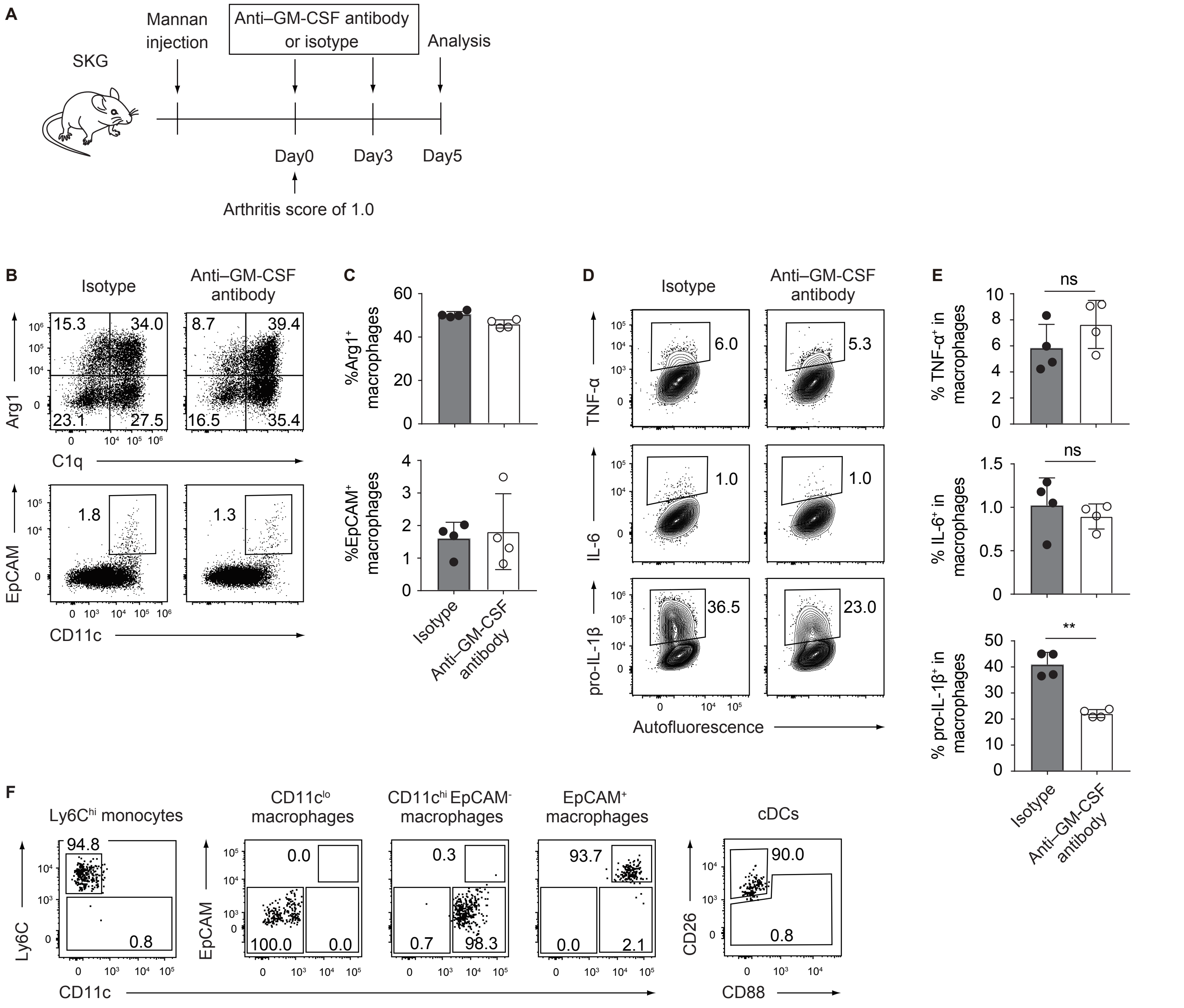

Figure S5. Anti-GM-CSF antibody treatment reduces pro-IL-1β production by synovial macrophages.

(A) Schematic of GM-CSF blockade experiment and analysis. Following mannan injection, SKG mice were intravenously administered either an anti–GM-CSF neutralizing antibody or an isotype control antibody on day 0 and day 3, when the arthritis score reached 1.0. Synovial macrophage subsets in inflamed joints were analyzed on day 5. (B) Flow cytometry analysis of Arg1 and C1q expression, along with surface markers EpCAM and CD11c, in CD11b^{+} Ly6G^{-} Ly6C^{lo} CD26^{-} synovial macrophages. (C) Percentages of Arg1^{+} and EpCAM^{+} macrophages in the synovial macrophages (n = 4 each). (D) Intracellular staining of TNF-α, IL-6, and pro-IL-1β in CD11b^{+} Ly6G^{-} Ly6C^{lo} CD26^{-} CD200^{-} synovial macrophages. (E) Percentages of TNF-α^{+}, IL-6^{+}, and pro-IL-1β^{+} macrophages (n = 4 each). (F) Flow cytometry analysis confirming the sorting purity of Ly6C^{hi} monocytes, EpCAM^{+} macrophages, CD11c^{lo} macrophages, CD11c^{hi} EpCAM^{-} macrophages and cDCs, as shown in Fig. 4H.

**$p < 0.01$. Statistical analyses were performed using Student' s t test (C and E). Error bars denote the SD in panels. Data in (B, D and F) are the representatives; data in (C and E) are pooled from two independent experiments.

Table S1. Gene lists used for scoring of MNPs

Proinflammatory cytokines	M2 macrophage signature genes
Il6	*Arg1*
Nlrp3	*Mrc1*
Il1b	*Retnla*
Tnf	*Cd163*
Ccl6	*Stat6*
Ccl1	*Irf4*
Ccl2	*Il10*
Ccl3	*Tgfb1*
Ccl4	*Fcgr3*
Ccl5	*Mertk*
Ccl6	*Hilpda*
Ccl7	
Ccl8	
Ccl9	
Ccl12	
Ccl17	
Ccl20	
Ccl22	
Ccl24	
Cxcl1	
Cxcl2	
Cxcl3	
Cxcl9	
Cxcl10	
Cx3cl1	
Il12b	
Il23a	
Csf1	
Csf3	
Ereg	
Il15	
Il18	
Il1a	
Lif	
Lta	
Osm	
